# Impact of old age on the association between in-center extended-hours hemodialysis and mortality in patients on incident hemodialysis

**DOI:** 10.1371/journal.pone.0235900

**Published:** 2020-07-10

**Authors:** Masaki Okazaki, Daijo Inaguma, Takahiro Imaizumi, Manabu Hishida, Shimon Kurasawa, Yoko Kubo, Sawako Kato, Yoshinari Yasuda, Takayuki Katsuno, Fumika Kaneda, Shoichi Maruyama

**Affiliations:** 1 Department of Nephrology, Nagoya University Graduate School of Medicine, Nagoya, Japan; 2 Department of Clinical Trials and Research, National Hospital Organization Nagoya Medical Center, Nagoya, Japan; 3 Department of Nephrology, Fujita Health University School of Medicine, Toyoake, Japan; 4 Department of Advanced Medicine, Nagoya University Hospital, Nagoya, Japan; 5 Center for Clinical Epidemiology and Biostatistics, University of Pennsylvania, Philadelphia, Pennsylvania, United States of America; 6 Department of Epidemiology, Johns Hopkins Bloomberg School of Public Health, Baltimore, Maryland, United States of America; 7 Department of Preventive Medicine, Nagoya University Graduate School of Medicine, Nagoya, Japan; 8 Department of Nephrology and Rheumatology, Aichi Medical University School of Medicine, Nagakute, Japan; 9 Kamome Clinic, Hitachi, Japan; Kaohsiung Medical University Hospital, TAIWAN

## Abstract

With the global problem of aging, it has become more difficult to improve the prognosis of older dialysis patients. Extended-hours hemodialysis offers longer treatment time compared to conventional hemodialysis regimen and provides favorable metabolic status, hemodynamic stability, and increased dietary intake. Despite prior studies reporting that in-center extended-hours hemodialysis can reduce the mortality rate, the treatment impact on elderly patients remains unclear. Therefore, we examined the association between extended-hours hemodialysis compared to conventional hemodialysis and all-cause mortality. Survival analyses using Cox proportional hazard model with multivariable adjustments and propensity-score based method were performed to compare mortality risk between 198 consecutive patients who started in-center extended-hours hemodialysis (Extended-HD) and 1407 consecutive patients who initiated conventional hemodialysis. The median age was 67.1 years in the Extended-HD group and 70.7 years in the conventional hemodialysis group. Extended-HD was associated with lower all-cause mortality in overall patients and the subgroup >70 years (adjusted hazard ratios of 0.60 [95% CI, 0.39–0.91] and 0.35 [95% CI, 0.18–0.69], respectively). There was a significant interaction between age >70 years and Extended-HD. In conclusion, extended-hours hemodialysis was associated with a lower mortality rate, especially in elderly patients.

## Introduction

As the world’s population ages, increasing numbers of elderly patients have been started on renal replacement therapy and are becoming a major part of the incident dialysis patient population. The incidence of end stage kidney disease (ESKD) is highest in the group over 75 years old in many developed countries. [1–3] Over the last decades, there has been growing concern that elderly patients with multiple comorbidities would have poor outcomes on dialysis. [4–6] Concerning peritoneal dialysis (PD), it is difficult for many elderly people to perform PD themselves, which puts a burden on the family caregivers. In the aging society of Japan, PD is performed by only 3% of dialysis patients. [7] Regarding kidney transplantation, there is increasing concern about perioperative complications and death, especially in frail elderly patients. [8, 9] In addition, the number of deceased kidney donations is highly limited in Japan. [10] Therefore, most elderly ESKD patients will default to hemodialysis (HD). Among the negative aspects of HD therapy, elderly patients can develop intradialytic hypotension and prolonged post-dialysis recovery time. [11–14] In order to provide better treatment for elderly patients, healthcare providers have attempted to customize HD therapy, such as longer dialysis sessions. Performing HD at home would be one option, which enables individuals to have control over the dialysis prescription. However, it needs supports from family or paid caregivers, who are not always available. Thus, there is a clinical need to provide customized in-center HD.

In the past decades, studies showed that in-center extended-hours HD was associated with improved blood pressure, anemia, and fluid management, and favorable metabolic parameters, as well as an increase in post-dialysis body weight. [15–17] Some observational studies have reported that in-center nocturnal HD, which provides substantially longer treatment length, was associated with lower mortality than conventional HD. [17, 18] However, the patients on in-center nocturnal HD in these studies were relatively young, with a mean age of under 60 years. Thus, it is still unclear whether in-center extended-hours HD improves survival for elderly dialysis patients with multiple comorbidities. Our prior study on extended-hours HD, based on the treatment policy of extending the dialysis time and removing dietary restrictions, reported an association between maintained or increased body mass index (BMI) and better survival. [19] Our hypothesis is that extended-hours HD without dietary restrictions improves survival in dialysis patients who are prone to malnutrition.

The aim of the present study was to determine the association between in-center daytime extended-hours HD and survival in incident HD patients. Mortality was compared between incident dialysis patients who exclusively initiated extended-hours HD and those who initiated conventional HD in the outpatient setting using a combined cohort of extended-hours HD and conventional HD patients. Whether the association between extended-hours HD and mortality differed in the elderly population was also evaluated.

## Materials and methods

### Study design and population

A retrospective cohort study of 198 consecutive incident dialysis patients who had exclusively received in-center extended-hours HD (Extended-HD) after the initiation of renal replacement therapy at 3 facilities operated by a specialized longer dialysis treatment provider located in the rural area of North-Eastern Japan during October 2008 to September 2017 was performed. All outpatients who voluntarily visited three specialized facilities were recommended by clinicians to be treated with daytime extended-hours HD. All consecutive outpatients who agreed to extended-hours HD treatment and treated for at least 3 months were included in this study. Extended-HD was defined as six or more hours per session, which is the most widely accepted threshold. [20] This study also included the Aichi Cohort Study of Prognosis in Patients Newly Initiated into Dialysis (AICOPP), the prospective cohort representing patients on conventional HD, in which all participants basically received HD of three to five hours per session three times per week. AICOPP enrolled 1407 consecutive incident HD patients in 17 facilities between October 2011 and September 2013 (Conventional HD). [21, 22] The baseline was defined as the time from after discharge to starting outpatient maintenance dialysis. Patients who died within 91 days after baseline or who had metastatic cancers were excluded. Patients on nocturnal HD were also excluded. Finally, the two dialysis cohorts were combined into an analytic cohort of 1553 patients. The present study was approved by the Institutional Review Boards of Nagoya University (approval no. 2014–0422) and the Fujita Health University (approval no. HM18-230), with an exemption from informed consent. All analytical data were fully anonymized before we accessed them.

### Outcome

The primary outcome was all-cause death. Mortality data and censoring events were obtained by medical chart review, telephone, or questionnaire from participating hospitals or dialysis facilities. Patients were followed from baseline up to the first 5 years, until death, or up to other censoring events, including kidney transplantation, recovery of kidney function, or transfer to another facility.

### Data collection

Covariates were variables suspected of being associated with either the anticipated treatment (i.e., obesity, diabetes, hypertension) or outcomes, based on clinical experience and evidence from the literature. All covariates were obtained from the electronic medical records accessed at the Kamome Clinic and the 17 AICOPP facilities [22] during April 2015 to March 2020. Collected data in this study included baseline patient characteristics: age, sex, BMI, serum albumin, hemoglobin, primary kidney disease (diabetic nephropathy, glomerulonephritis, hypertensive disease, others), comorbid conditions (diabetes, coronary heart disease, peripheral artery disease, aortic disease, cerebrovascular disease, cardiovascular disease [any of the following: coronary heart disease, peripheral artery disease, aortic disease, and cerebrovascular disease], liver disease, and malignancy), Charlson comorbidity index, vascular access (arteriovenous fistula or graft, long-term central venous catheter, and others including superficialization of the artery [23] or direct venipuncture [24]), and antihypertensive medication use. BMI was calculated using post-dialysis body weight at the baseline. The Charlson comorbidity index was calculated using a combined age-comorbidity score including the kidney disease score. [25] In order to describe the characteristics of the Extended-HD regimen, individual dialysis session records were obtained to clarify the parameters of the administered dialysis treatment. Laboratory data measured at the beginning of the week were obtained closest to the first 91-day phase after enrollment. Single-pool Kt/V urea was measured twice a year, and the closest Kt/V values were obtained after the 91-day point from baseline. To minimize measurement variability, treatment parameters during the two weeks before and after the 91-day point were averaged for exposure description.

### Statistical analysis

Baseline characteristics of the analytic cohort were summarized by treatment group and are presented as medians (IQR) for continuous variables and percentages for categorical variables. Baseline characteristics among the treatment groups were assessed using the Mann-Whitney *U* test for continuous variables and the chi-squared test for categorical variables. Kaplan-Meier analysis with log-rank tests was used for the survival analysis of the treatment groups and age subgroups (> or ≤70 years). Crude rates for all-cause mortality were calculated, and these crude mortalities stratified by age subgroups were also calculated. Associations between treatment groups and all-cause mortality were analyzed using a Cox proportional hazards model. We also examined each cause of death using competing risk analysis with the cause-specific proportional hazards model. Proportional hazards assumptions were tested by log-log plots and by Schoenfeld residuals. The listed covariates were entered into the multivariable Cox model 1: age, sex, BMI category, diabetes, cardiovascular disease, liver disease, malignancy, vascular access, and class of antihypertensive agents. Model 2 consisted of variables in model 1 plus serum albumin and hemoglobin. Sensitivity analyses were performed to evaluate the robustness of the main findings. To confirm the estimated effects of Extended-HD obtained by the multivariable analyses, the data were further analyzed by propensity score-based methods. Propensity scores were calculated for each patient with Extended-HD as the dependent variable using age, sex, BMI category, serum albumin, hemoglobin, primary kidney diseases, comorbid conditions, Charlson comorbidity index, vascular access, and class number of antihypertensive agents. PS-matching was performed for balancing the baseline characteristics of Extended-HD and Conventional HD using a nearest-neighbor 1:4 matching. PS-stratification technique consisted of allocating patients to five strata according to quintiles of their propensity scores, and the obtained scores were included in a Cox proportional hazards model as a linear covariate. The associations of Extended-HD with all-cause mortality were examined by stratified analyses in the subgroups of age, sex, presence or absence of diabetes, cardiovascular disease, and BMI category. Variables relevant to the subgroups were excluded from each model. Potential interactions were formally tested by likelihood-ratio tests. A two-tailed P-value < 0.05 was considered statistically significant in all analyses. Statistical analyses were conducted using Stata MP version 15.1 (Stata Corp., College Station, TX, USA).

## Results

### Baseline characteristics

**Fig 1:**
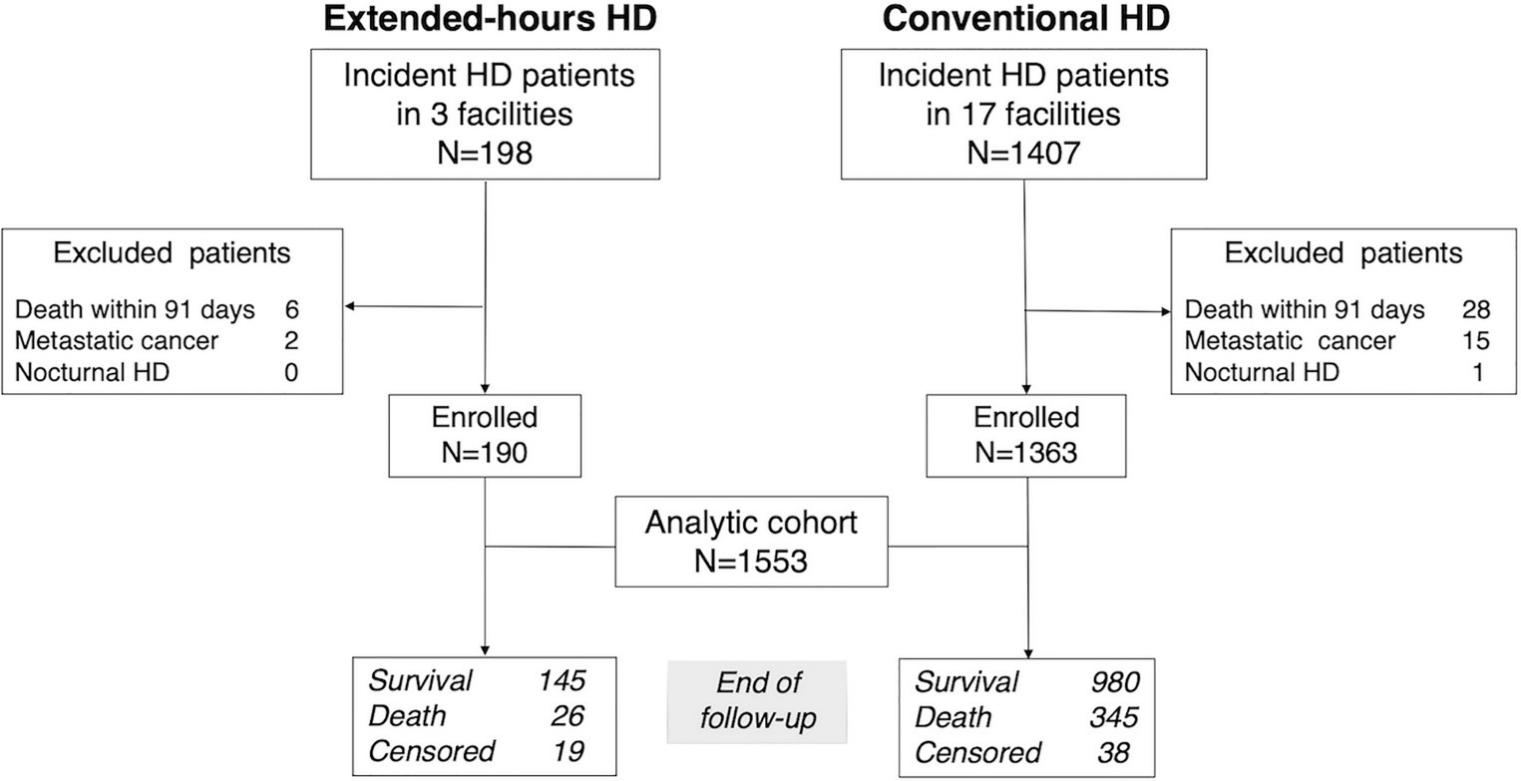
Flow diagram of participants constituting the analytic cohort.

The analytic cohort of 1,553 patients consisted of 190 incident dialysis patients treated with Extended-HD and 1,363 incident patients treated with Conventional HD as the control group (Fig 1). The median age of the entire cohort was 70.2 (interquartile range [IQR], 60.9–77.8) years. Table 1 shows that the Extended-HD group was younger (67.1 [IQR, 54.7–75.5] vs. 70.7 [IQR 62.1–78.0] years) and had a higher prevalence of diabetes (63% vs. 55%) compared to the Conventional HD group. In the age subgroups stratified by age > or ≤70 years, the proportion of male sex, diabetes, peripheral artery disease, and liver disease were higher in the Extended-HD group ≤70 years of age. In the patients >70 years of age, there were no significant differences between the treatment groups. Regarding vascular access, there were no cases of use of a long-term central venous catheter in both groups. Overall, 7% of the Extended-HD group had superficialization of an artery or direct venipuncture, whereas 11% of the Conventional HD group had an arteriovenous graft placed. Of the patients treated with Extended-HD, nineteen patients transferred to another facility providing conventional HD during follow-up periods. Of the patients treated with Conventional HD, sixteen patients received kidney transplantation, sixteen patients transferred to another facility, six patients recovered kidney function.

**Table 1:** Baseline characteristics of patients stratified by treatment group and age subgroups.

	Overall		Age subgroups			
			Age >70 years		Age ≤70 years	
	Extended-hours HD (n = 190)	Conventional HD (n = 1363)	Extended-hours HD (n = 73)	Conventional HD (n = 714)	Extended-hours HD (n = 117)	Conventional HD (n = 649)
**Demographics**						
Age, years	67.1 [54.7–75.5]	70.7 [62.1–78.0]†	77.5 [74.9–81.4]	77.7 [73.9–81.9]	58.4 [50.1–65.7]	61.2 [51.4–65.4]
Male sex	73%	67%	63%	66%	79%	69%‡
BMI, kg/m^2^	23.1 [21.0–25.7]	22.6 [20.4–25.1]‡	22.5 [20.2–23.8]	21.8 [19.8–23.9]	24.1[21.5–27.1]	23.7 [21.2–26.7]
BMI category						
BMI >25 kg/m^2^	33%	27%	12%	13%	43%	38%
18.5< BMI ≤25 kg/m^2^	60%	63%	71%	71%	52%	54%
BMI ≤18.5 kg/m^2^	7%	10%	17%	16%	5%	8%
Laboratory data*						
Serum albumin, g/dL	3.3 [3.0–3.7]	3.2 [2.8–3.6]†	3.4 [3.1–3.7]	3.2 [2.8–3.5]†	3.3 [2.9–3.6]	3.2 [2.8–3.6]
Hemoglobin, g/dL	9.4 [8.4–10.1]	9.4 [8.3–10.3]	9.4 [8.8–10.1]	9.4 [8.4–10.3]	9.4 [8.3–10.1]	9.4 [8.3–10.4]
**Primary kidney diseases**						
Diabetic nephropathy	53%	44%	33%	36%	65%	53%
Glomerulonephritis	13%	14%	11%	11%	14%	17%
Hypertensive disease	21%	26%	39%	39%	8%	13%
Others	14%	16%	17%	14%	13%	17%
**Comorbid conditions**						
Diabetes	63%	55%‡	48%	52%	73%	59%†
Coronary heart disease	15%	17%	21%	22%	11%	11%
Peripheral artery disease	8%	5%	3%	6%	12%	5%†
Aortic disease	6%	6%	11%	8%	2%	3%
Cerebrovascular disease	18%	16%	16%	19%	17%	13%
Cardiovascular disease	32%	37%	37%	45%	29%	27%
Liver disease	7%	4%	6%	5%	8%	4%
Malignancy	5%	6%	7%	8%	3%	3%
Charlson comorbidity index	6 [5–8]	6 [5–8]‡	8 [6–9]	7 [6–8]‡	6 [4–7]	5 [4–6]†
**Vascular access**						
Arteriovenous fistula	93%	88%	92%	86%	93%	92%
Arteriovenous graft	0%	11%	0%	13%	0%	8%
Central venous catheter	0%	0%	0%	0%	0%	0%
Others	7%	1%†	8%	1%†	7%	0%†
**Antihypertensive medication**						
ACE inhibitor or ARB	70%	60%†	74%	57%†	69%	64%
Calcium-channel blocker	82%	79%	88%	79%	79%	78%
β-Blocker	31%	35%	36%	37%	28%	33%
Aldosterone antagonist	0%	5%†	0%	4%	0%	7%†
Antihypertensive drug classes						
0	9%	9%	4%	8%	12%	10%
1–2	58%	57%	63%	58%	55%	56%
≥3	33%	34%	33%	34%	33%	34%

Values for continuous data are shown as medians [interquartile range] for non-normally distributed data.

Abbreviations: BMI, body mass index; ACE, angiotensin-converting enzyme; ARB, angiotensin-receptor blocker

*A total of 145 (76.3%) participants in Extended-hours HD had available data for both serum albumin and hemoglobin, and a total of 1361 (99%) participants in Conventional HD had available data for both serum albumin and hemoglobin.

^†^: P<0.01 for the comparison between the Extended-hours HD and Conventional HD groups.

^‡^: P<0.05 for the comparison between the Extended-hours HD and Conventional HD groups.

Regarding Extended-HD during the first 91-day period after enrollment, the average dialysis length was 6–7 hours per session, and approximately 98% had a thrice-weekly treatment frequency (S1 Table). The patients on Extended-HD were treated in the daytime or finished the session by midnight. The median blood flow rate was 130 [IQR, 120–150] ml/min, and thus the median value of single-pool Kt/V urea was 1.3 [IQR, 1.1–1.6].

### Extended-hours hemodialysis and all-cause mortality

**Fig 2:**
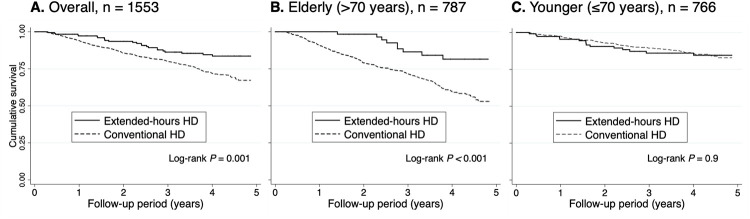
(A, B and C). Kaplan-Meier analysis of all-cause mortality stratified by age subgroup comparing the extended-hours HD and conventional HD groups.

During a median follow-up period of 3.7 years for the Extended-HD group and 3.5 years for the Conventional HD group, a total of 371 deaths were observed. Extended-HD showed better outcomes than Conventional HD overall and for the subgroup >70 years of age on Kaplan-Meier analysis (log-rank P = 0.001, P < 0.001, respectively) (Fig 2). For the sensitivity analysis, we conducted 1:4 PS-matched cohort (S2 Table). The imbalances in baseline characteristics of entire cohort were balanced after propensity score matching, with each standardized difference in absolute value of <0.1. In the PS-matched cohort, Kaplan-Meier analysis similarly showed better survival of Extended-HD overall and for the subgroup of >70 years old (log-rank P = 0.006, P < 0.001, respectively) (S1 Fig). Table 2 shows the mortality rate and adjusted hazard ratio (HR) for all-cause death with Extended-HD using multivariable-adjusted Cox proportional hazards models. Crude mortality rate was 4.1 deaths per 100 patient-years in the Extended-HD group compared with 7.8 deaths per 100 patient-years in the Conventional HD group. Adjusted HRs of Extended-HD in overall patients and the subgroup >70 years of age were 0.60 [95% confidence interval (CI), 0.39–0.91] and 0.35 [95% CI, 0.18–0.69] in multivariable model 1, respectively. Similar associations were observed in multivariable model 2. The propensity score-adjusted Cox proportional hazards models for Extended-HD showed a lower risk of death in overall patients and in the subgroup aged >70 years (1:4 Matched: HRs of 0.49 [95% CI, 0.28–0.84] and 0.25 [95% CI, 0.09–0.69], respectively). In the subgroup aged ≤70 years, the association between Extended-HD and mortality was not significant in multivariable adjustment (model 1: HR of 0.95 [95% CI, 0.54–1.65]).

**Table 2:** Mortality rate and hazard ratios for all-cause mortality stratified by age subgroups.

Characteristics	n	Patient-year	Mortality^a^	Multivariable-adjusted		Propensity score adjustment^d^	
				Model 1^b^ (n = 1553)	Model 2^c^ (n = 1507)	Matched (1:4, n = 710)	Stratification (n = 1502)
				HR (95% CI)	HR (95% CI)	HR (95% CI)	HR (95% CI)
**Overall**							
Conventional HD	1363	4411	7.8	1.00 (reference)	1.00 (reference)	1.00 (reference)	1.00 (reference)
Extended-hours HD	190	638	4.1	0.60 (0.39–0.91)*	0.56 (0.33–0.94)*	0.49 (0.28–0.84)*	0.47 (0.28–0.80)*
**Age >70 year**							
Conventional HD	714	2172	12.0	1.00 (reference)	1.00 (reference)	1.00 (reference)	1.00 (reference)
Extended-hours HD	73	238	4.2	0.35 (0.18–0.69)*	0.25 (0.09–0.67)*	0.25 (0.09–0.69)*	0.21 (0.08–0.56)*
**Age ≤70 years**							
Conventional HD	649	2239	3.8	1.00 (reference)	1.00 (reference)	1.00 (reference)	1.00 (reference)
Extended-hours HD	117	400	4.0	0.95 (0.54–1.65)	0.97 (0.51–1.86)	0.85 (0.44–1.63)	1.04 (0.55–1.97)

Abbreviations: HR, hazard ratio; CI, confidence interval.

^a^Crude mortality rate per 100 patient-years.

^b^Model 1: adjusted for age, sex, body mass index, diabetes, cardiovascular disease, malignancy, liver disease, vascular access, and class of antihypertensive agents.

^c^Model 2: adjusted for variables in model 1 plus serum albumin and hemoglobin.

^d^Propensity scores were calculated for each patient with extended-hours hemodialysis as the dependent variables using age, sex, body mass index, primary kidney diseases, comorbid conditions, Charlson comorbidity index, vascular access, class number of antihypertensive agents, serum albumin, and hemoglobin. The propensity-score stratification technique consisted of allocating patients to five strata according to quintiles of their propensity scores. The estimated propensity scores were included in a Cox proportional hazards model as a linear covariate.

*P < 0.05.

### Effects of age and several potential factors on the association between extended-hours HD and mortality

**Fig 3:**
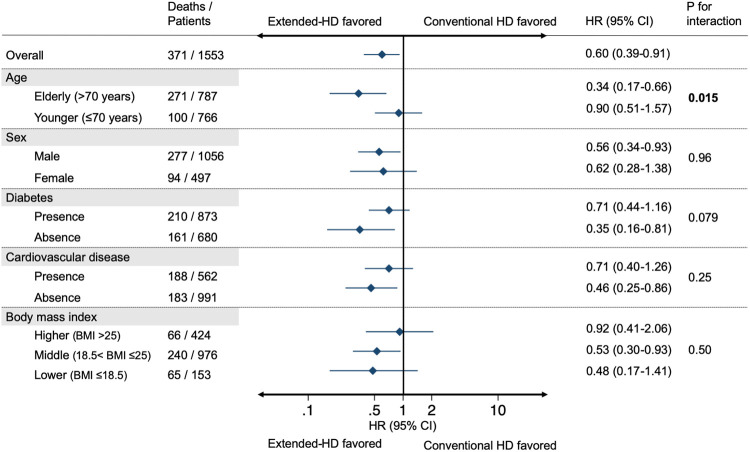
Multivariable-adjusted hazard ratios and 95% confidence intervals for the association of extended-hours hemodialysis on all-cause mortality stratified by potential important confounders. Results were adjusted for age, sex, category of body mass index, diabetes, cardiovascular disease, liver disease, malignancy, vascular access, and class of antihypertensive agents. Variables relevant to the subgroups were excluded from each model. Abbreviations: HR, hazard ratio; CI, confidence interval.

In the multivariable-adjusted Cox models, the low HRs for all-cause mortality in the Extended-HD group were consistent across the clinical subgroups (Fig 3). The association was significant in some subgroups: HRs of 0.34 (95% CI, 0.17–0.66) in the elderly population (>70 years old), 0.56 (95% CI, 0.34–0.93) in the male sex subgroup, 0.35 (95%CI, 0.16–0.81) in the non-diabetes subgroup, and 0.46 (95%CI, 0.25–0.86) in the non-cardiovascular disease subgroup. The effect of BMI on the association between Extended-HD and mortality showed a dose-response relationship (adjusted HRs of 0.48 [95% CI, 0.17–1.41] and 0.53 [95% CI, 0.30–0.93] in the lowest and the middle groups vs. 0.92 [95% CI, 0.41–2.06] in the highest group). In the subgroup of BMI ≤18.5 kg/m^2^, the prevalence of patients over 70 years old was 65%. The interaction between elderly population (aged >70 years) and Extended-HD was significant (P for interaction 0.015). In the sensitivity analysis, the significant associations with low mortality in the Extended-HD group were found in subgroups of elderly, male sex, non-diabetes, non-cardiovascular disease, and middle group of BMI in the PS-matched cohort (S2 Fig).

**Fig 4:**
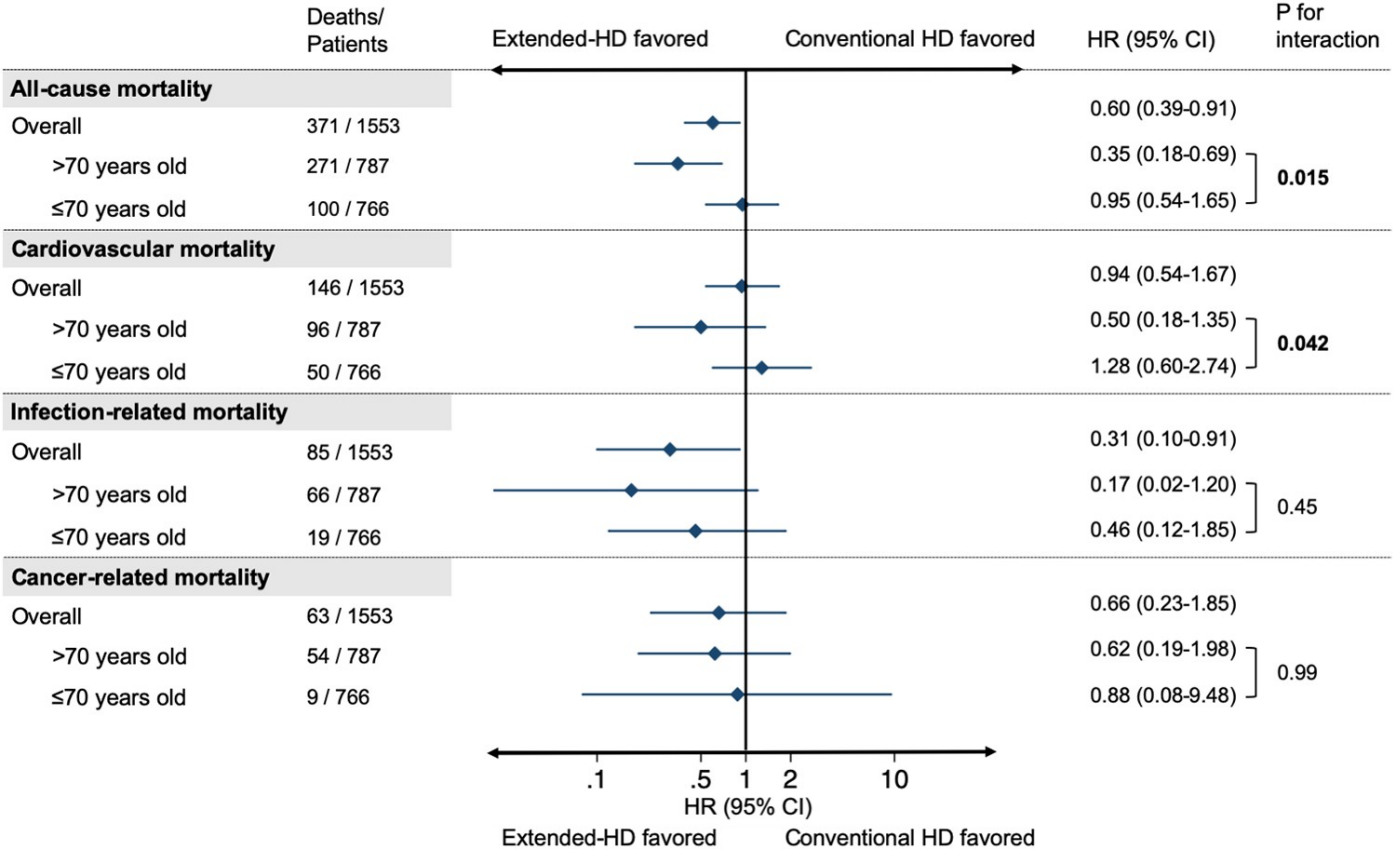
Adjusted hazard ratios and 95% confidence intervals for each cause of death in the Extended-HD group compared to the conventional HD group stratified by age subgroups. The estimated risks were adjusted for age, sex, category of body mass index, diabetes, cardiovascular disease, liver disease, malignancy, vascular access, and class of antihypertensive agents. Abbreviations: HD, hemodialysis; HR, hazard ratio; CI, confidence interval.

Regarding the causes of death, Extended-HD was associated with lower risk of infection-related mortality than Conventional HD using competing risk analysis. A similar tendency was observed in both age subgroups over or under 70 years old (Fig 4, S3 Table).

## Discussion

This retrospective cohort of incident dialysis patients clearly demonstrated that in-center daytime extended-hours HD was associated with a lower risk of death than conventional HD. This association was consistent after accounting for basic demographics and clinically relevant factors. The association between extended-hours HD and mortality was especially prominent in elderly patients aged over 70 years. In contrast, in patients ≤70 years, there was no significant association with mortality. To the best of our knowledge, this is the first study to show the lower risk of mortality in the elderly population undergoing extended-hours HD.

Previous observational studies have reported that in-center extended-hours HD performed overnight was associated with better survival than conventional HD. [17, 18] However, the nocturnally treated patients enrolled in these prior studies were relatively young, with mean ages of 45.2 and 52.3 years, respectively. These experimental population in previous randomized trials may not include a sensitive population that would benefit from extended dialysis time and accompanying increased dietary intake. Thus, there is still limited evidence of the survival benefit of extended-hours hemodialysis in real-world practice today. No studies have shown that in-center extended-hours HD is associated with improved survival for patients with an average age over 60 years. The present study, therefore, focused on incident patients treated during the daytime with a median age of 70.2 years, which was similar to the mean age of 69.2 years in incident dialysis patients in Japan’s national registry in 2015. [3] Based on the recent Japanese demographics, a stratified analysis was performed with a threshold of 70 years. In the present study, the demographics and comorbid conditions of enrolled patients older than 70 years were quite similar between the Extended-HD and Conventional HD groups. The cohort of Extended-HD was representative of regional, general dialysis patients registered in the rural area of North-Eastern Japan. Because the number of hospitals per 100,000 population is below the average in these provinces, [26] geographic factors, rather than treatment policy, would have been strong in choosing a dialysis facility, especially among older patients. Actually, 98% of the recruited patients in the Extended-HD group visited the facilities from within the cities or adjacent townships. The patients over the age of 70 in Extended-HD group were attending the specialized Extended-HD facilities simply because the facilities were close to the patient’s homes and many elderly patients usually used the pickup transportation service. Therefore, the patient backgrounds especially in aged >70 years was similar between two treatment groups. The Extended-HD patients aged <70 years had significantly more diabetic men with peripheral artery disease. There may be a selection bias for Extended-HD patients recommended by the attending physician before starting maintenance dialysis, who was concerned about the high risk of death or hospitalization due to coexisting severe comorbidities especially when the patients were relatively young.

There were three therapeutic features of Extended-HD in the present study. The first feature was a long dialysis treatment time, which leads to a lower ultrafiltration rate and reduced intradialytic hypotension. [12, 17, 18] Rapid fluid removal is associated with greater risks of mortality and cardiovascular events, [27] and it may cause intradialytic hypotension, which is also associated with higher mortality. [28] Notably, there is a strong association between hypotensive events and advancing age. [11, 29] Thus, stability of hemodynamics is a particular concern in elderly dialysis patients. The second feature was a lower blood flow rate. In order to minimize post-dialysis fatigue, the blood flow rates were kept intentionally low at a median value of 130 mL/min, satisfying KDOQI minimum Kt/V levels. [30] The third feature was the extended dialysis time without dietary restrictions, which may lead to increased nutritional intake. In fact, the staff encouraged patients to consume normal meals with their families, rather than undergo dietary restrictions, and increase their body weight. In Japan, there are growing concerns about strict dietary restrictions during the non-dialysis CKD and the spread of malnutrition at the initiation of maintenance hemodialysis. High rates of malnutrition, recently described as protein-energy wasting, have been reported in the area of dialysis care, and malnutrition is associated with mortality and morbidities. [31, 32] In fact, BMI or lean body mass tends to decrease in maintenance HD patients. [33–35] In contrast, prior observational studies showed that in-center nocturnal HD was associated with an increased post-HD weight, which is considered one of the indirect indicators of nutritional status. [17, 18] In addition, our recent study reported that the majority of patients who received extended-hours HD without dietary restrictions showed BMI increases. [19] A possible mechanism that underlies the effects of extended-hours HD with no dietary restrictions is improvement in malnutrition, which may be associated with host immunity. The present study showed a reduced risk for infection-related mortality in the Extended-HD group compared to the Conventional HD group. Malnutrition is a common cause of secondary immune deficiency and susceptibility to infection in humans. [36] In fact, a recent study based on the Japanese nationwide registry for dialysis data reported that dialysis patients with a low nutritional status show a high risk of death due to infection. [37] We believe that the treatment policies of extending the time of the dialysis session and no dietary restrictions can improve the nutritional status, and may consequently reduce mortality in frail elderly patients on chronic HD.

There is a need to discuss why Extended-HD was beneficial for elderly patients. In the present stratified analysis, there was a dose-response relationship of BMI categories in the association between Extended-HD and mortality, with the lowest HR in the BMI ≤18.5 kg/m^2^ category (Fig 3). Importantly, 65% of the patients in this subgroup of BMI ≤18.5 kg/m^2^ were elderly patients aged >70 years. These findings may provide one of the reasons why the elderly patients were more likely to benefit from the treatment policy for extended-hours HD. In patients with ESKD, inverse relationship between high BMI and better survival has been consistently found, which is unlikely to be due to residual confounding alone and has biologic plausibility. [38, 39] The rationale to avoid underdialysis may be more reasonable for elderly patients because uremic intoxication could be more important in older patients as the ratio between the metabolic compartment producing the toxins and the diffusion space of these toxins is higher in patients with low BMI. [40] In terms of uremic sarcopenia, elderly patients are particularly susceptible to uremic state and this accelerates the physiological uremic muscle wasting. [41] Our previous study based on the same treatment policy reported an association between maintained or increased BMI and reduced risk of mortality, [19] which supports the importance of considering nutritional status in elderly population. We should consider another possibility, that a higher prevalence of diabetes in the younger subgroup aged ≤70 years affected their prognosis. We need to be careful when we encourage patients with diabetes to eat without dietary restrictions, since it might cause adverse consequences (e.g., deterioration of glycemic control or diabetic complications). Although the detailed information about glycemic control was lacking, prior studies reported an association between uncontrolled hyperglycemia and adverse outcomes in HD patients. [42–44] Further studies are needed to assess the benefit of extended-hours HD without dietary restrictions taking into account age and diabetic status.

There were several limitations in this study. First, there would have been selection bias of patient’s preference that could not be completely adjusted with the propensity score method. Due to the selection bias, the association may be overestimated. To minimize the selection bias, we targeted in-center incident dialysis patients treated with daytime therapy, not nocturnal patients. Second, dialysis prescription data and dialysis adequacy on conventional HD were not available. Longitudinal data on dialysis prescription, echocardiography, and mineral bone/metabolism factors were also lacking. Reviewing recent literature based on The Dialysis Outcomes and Practice Patterns Study (DOPPS) data, which is a representative dialysis cohort in Japan, the small molecule clearance on Extended-HD was similar to that of the DOPPS cohort (S1 Table). [45] Third, due to the nature of an observational study, residual confounding and unmeasured confounders (e.g., socioeconomic status, facility level confounders) could not be eliminated. Baseline creatinine levels at exactly the same laboratory testing were also not available. Fourth, because this study used a pooled cohort of two distinctive regional cohorts, we did not directly compare treatment groups with the same observation period and treatment environment. In Japan, regional differences are limited to differences in medical treatment levels and patterns, and there is little need to consider differences in races and health insurance system. The harmonization of variables in the treatment groups was not necessarily perfect, but on the other hand, there is an advantage that there was no crossover of patients between Extended-HD group and Conventional HD group. Finally, detailed information on the elements of the diagnostic criteria for protein-energy wasting were not available.

In the present study, in-center daytime extended-hours HD was associated with a lower risk of mortality than conventional HD. This relationship was especially prominent in elderly patients aged over 70 years. Well-designed prospective studies are needed to verify our hypothesis that the treatment policy of extending dialysis time without dietary restrictions improves survival in patients with ESKD.

## Supporting information

S1 Table Treatment parameters and laboratory data during the first 91 days in patients who received extended-hours hemodialysis. (DOCX)

S2 Table Baseline characteristics of extended-hours HD patients, propensity score-matched conventional HD patients, and entire conventional HD patients. (DOCX)

S3 Table Adjusted hazard ratios for cause-specific mortality in the Extended-HD group compared to the conventional HD group stratified by age subgroup. (DOCX)

S1 Fig Kaplan-Meier curves for all-cause mortality comparing the extended-hours HD and propensity score-matched conventional HD groups stratified by age subgroup. Patient characteristics of 1:4 matched cohort was shown in S1 Table. The median follow-up periods were 3.8 years for the Extended-hours HD group and 3.5 years for the Conventional HD group, respectively.
(TIFF)

S2 Fig Propensity score-matched hazard ratios and 95% confidence intervals for the association of extended-hours hemodialysis on all-cause mortality stratified by potential important confounders. Propensity scores were calculated using age, sex, body mass index, primary kidney diseases, comorbid conditions, charlson comorbidity index, vascular access, and class number of antihypertensive agents. Abbreviations: DM, diabetes mellitus; CVD, cardiovascular disease; BMI, body mass index.
(TIFF)
