# Supplementary material for: Impact of old age on the association between in-center extended-hours hemodialysis and mortality in patients on incident hemodialysis
Source: PLoS One. 2020 Jul 10;15(7):e0235900. doi: 10.1371/journal.pone.0235900 (PMC7351168; doi:10.1371/journal.pone.0235900)
Supplement: S1 Table — (DOCX) [file pone.0235900.s001.docx]

**S1 Table. Treatment parameters and laboratory data during the first 91 days in patients who received extended-hours hemodialysis**

| Characteristics of extended-hours HD | N | Percentage or Median [IQR] |
| --- | --- | --- |
| **Treatment parameters** | | |
| Dialysis length per sessions, hours | 188 | 6.0 [6.0-7.0] |
| Percentage of thrice weekly dialysis, % | 188 | 98% |
| Blood flow rate, mL/min | 183 | 130 [120-150] |
| Dialysate flow rate, mL/min | 183 | 300 [300-300] |
| ^a^Relative interdialytic weight gain, % | 183 | 3.2 [2.1-4.6] |
| Ultrafiltration rate, mL/hour | 187 | 400 [280-580] |
| **Laboratory data^b^** | | |
| Serum albumin, g/dL | 191 | 3.6 [3.3-3.8] |
| Serum creatinine, md/dL | 189 | 6.3 [4.9-8.0] |
| Hemoglobin, g/dL | 191 | 10.1 [9.3-10.9] |
| Adjusted serum calcium, mg/dL | 191 | 8.5 [8.2-9.0] |
| Serum phosphorus, mg/dL | 191 | 5.0 [4.2-6.3] |
| Serum alkaline phosphatase, U/L | 191 | 247 [204-307] |
| Serum parathyroid hormone (intact assay), pg/mL | 191 | 176 [109-261] |
| Single-pool Kt/V urea | 174 | 1.3 [1.1-1.6] |

Note: Each values are median and interquartile range, or proportions (%).

^a^The value was described as the percentage of post-dialysis body weight averaged weekly.

^b^Laboratory tests were examined immediately before the regular dialysis session at the beginning of the week.
