# Supplementary material for: Impact of old age on the association between in-center extended-hours hemodialysis and mortality in patients on incident hemodialysis
Source: PLoS One. 2020 Jul 10;15(7):e0235900. doi: 10.1371/journal.pone.0235900 (PMC7351168; doi:10.1371/journal.pone.0235900)
Supplement: S2 Table — (DOCX) [file pone.0235900.s002.docx]

**S2 Table. Baseline characteristics of extended-hours HD patients, propensity score-matched conventional HD patients, and entire conventional HD patients**

|  | **Extended-hours HD** | **Conventional HD** | | | |
| --- | --- | --- | --- | --- | --- |
|  |  | 1:4 Matched | Std Diff | Entire | Std Diff |
| Number of patients | 187 | 749 |  | 1363 |  |
| Age, years | 67.1  [54.7-75.5] | 67.4  [55.8-76.7] | -0.03 | 70.7  [62.1-78.0] | -0.27 |
| Male sex | 72% | 75% | -0.06 | 67% | 0.11 |
| BMI, kg/m^2^ | 23.1  [21.0-25.7] | 22.9  [20.7-25.7] | 0.04 | 22.6  [20.4-25.1] | 0.14 |
| **BMI category** |  |  | 0.05 |  | 0.14 |
| BMI >25 kg/m^2^ | 33% | 30% |  | 27% |  |
| 18.5< BMI ≤25 kg/m^2^ | 59% | 62% |  | 63% |  |
| BMI ≤18.5 kg/m^2^ | 8% | 8% |  | 10% |  |
| **Primary kidney diseases** |  |  | -0.06 |  | -0.16 |
| Diabetic nephropathy | 54% | 52% |  | 44% |  |
| Glomerulonephritis | 13% | 11% |  | 14% |  |
| Hypertensive disease | 19% | 22% |  | 26% |  |
| Others | 14% | 15% |  | 16% |  |
| **Comorbid conditions** |  |  |  |  |  |
| Diabetes | 64% | 61% | 0.07 | 55% | 0.17 |
| Coronary heart disease | 15% | 12% | 0.08 | 17% | -0.06 |
| Peripheral artery disease | 8% | 7% | 0.06 | 5% | 0.13 |
| Aortic disease | 5% | 4% | 0.06 | 6% | -0.03 |
| Cerebrovascular disease | 17% | 16% | 0.008 | 16% | 0.02 |
| Cardiovascular disease | 33% | 34% | -0.04 | 37% | -0.09 |
| Liver disease | 6% | 7% | -0.04 | 4% | 0.11 |
| Malignancy | 4% | 3% | 0.06 | 6% | -0.08 |
| Charlson comorbidity index | 6 [5-8] | 6 [5-8] | 0.03 | 6 [5-8] | 0.18 |
| **Vascular access** |  |  |  |  |  |
| Arteriovenous fistula | 93% | 95% | -0.07 | 88% | 0.17 |
| **Antihypertensive drug classes** |  |  | -0.05 |  | -0.03 |
| 0 | 9% | 9% |  | 9% |  |
| 1-2 | 58% | 55% |  | 57% |  |
| ≥3 | 33% | 36% |  | 34% |  |

Note: Values for continuous data are shown as medians [interquartile range] or percentage, appropriately. Standardized difference in absolute value of <0.1 is considered a negligible difference between groups. Propensity scores were calculated using age, sex, body mass index, primary kidney diseases, comorbid conditions, charlson comorbidity index, vascular access, and class number of antihypertensive agents.

Abbreviations: HD, hemodialysis; BMI, body mass index; Std Diff, standardized difference
