# Supplementary material for: Impact of old age on the association between in-center extended-hours hemodialysis and mortality in patients on incident hemodialysis
Source: PLoS One. 2020 Jul 10;15(7):e0235900. doi: 10.1371/journal.pone.0235900 (PMC7351168; doi:10.1371/journal.pone.0235900)
Supplement: S3 Table — (DOCX) [file pone.0235900.s003.docx]

**S3 Table. Adjusted hazard ratios for cause-specific mortality in the Extended-HD group compared to the Conventional HD group stratified by age subgroup**

|  | | Deaths/patients (%) | | HR (95% CI) |  |
| --- | --- | --- | --- | --- | --- |
|  |  | Extended-HD | Conventional HD | Cause-specific proportional hazards model^a^ | P for interaction |
| **Cardiovascular death** | |  |  |  |  |
| Overall | | 14/190 (7.3%) | 132/1363 (9.7%) | 0.94 (0.54-1.67) |  |
|  | Age >70 years | 4/73 (5.5%) | 92/714 (12.9%) | 0.50 (0.18-1.35) | **0.041** |
|  | Age ≤70 years | 10/117 (8.5%) | 40/649 (6.2%) | 1.28 (0.60-2.74) |  |
| **Infection-related death** | |  |  |  |  |
| Overall | | 3/190 (1.6%) | 82/1363 (6.0%) | 0.31 (0.10-0.91) |  |
|  | Age >70 years | 1/73 (1.4%) | 65/714 (9.1%) | 0.17 (0.02-1.20) | 0.45 |
|  | Age ≤70 years | 2/117 (1.7%) | 17/649 (2.6%) | 0.46 (0.12-1.85) |  |
| **Cancer-related death** | |  |  |  |  |
| Overall | | 4/190 (2.1%) | 59/1363 (4.3%) | 0.66 (0.23-1.85) |  |
|  | Age >70 years | 3/73 (4.1%) | 51/714 (7.1%) | 0.62 (0.19-1.98) | 0.99 |
|  | Age ≤70 years | 1/117 (0.9%) | 8/649 (1.2%) | 0.88 (0.08-9.48) |  |

^a^Multivariable adjustment was performed using age, sex, category of body mass index, diabetes, cardiovascular disease, liver disease, malignancy, vascular access, and class of antihypertensive agents.

Abbreviations: HD, hemodialysis; HR, hazard ratio; CI, confidence interval
