# Supplementary figures and images for: Impact of old age on the association between in-center extended-hours hemodialysis and mortality in patients on incident hemodialysis
Source: PLoS One. 2020 Jul 10;15(7):e0235900. doi: 10.1371/journal.pone.0235900 (PMC7351168; doi:10.1371/journal.pone.0235900)

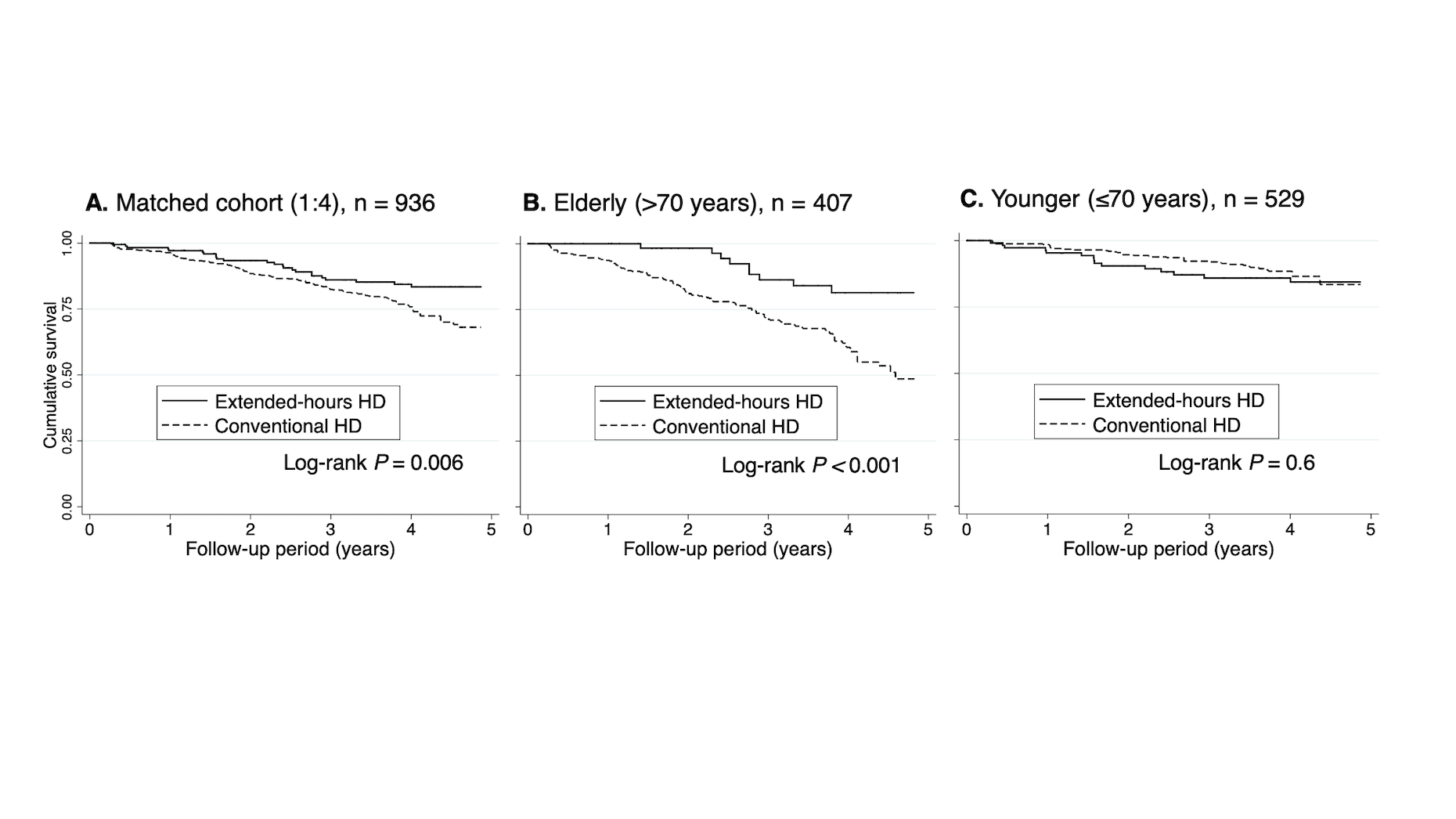

Supplement: S1 Fig — Patient characteristics of 1:4 matched cohort was shown in S1 Table. The median follow-up periods were 3.8 years for the Extended-hours HD group and 3.5 years for the Conventional HD group, respectively. (TIFF) [file pone.0235900.s004.tiff]

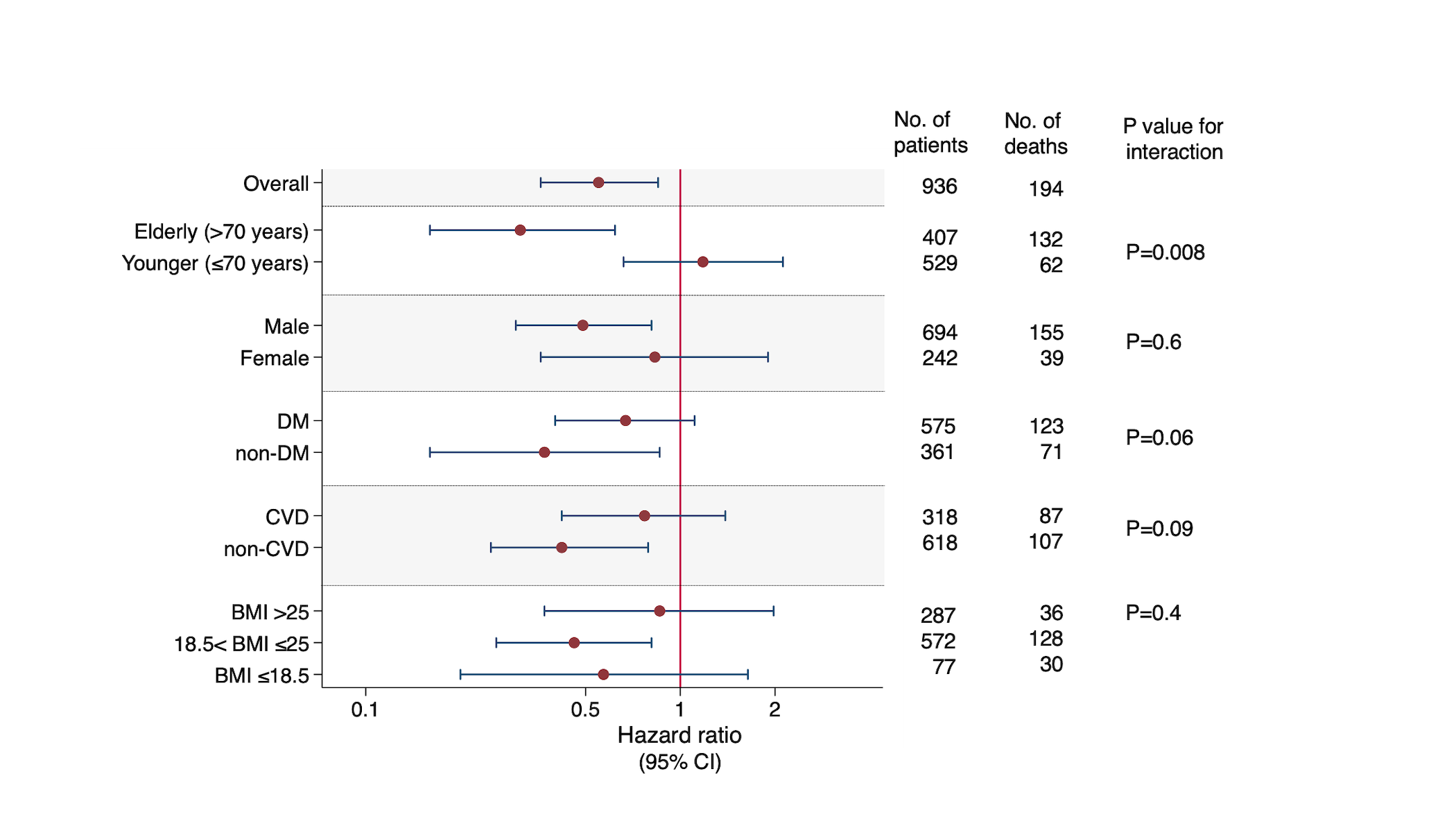

Supplement: S2 Fig — Propensity scores were calculated using age, sex, body mass index, primary kidney diseases, comorbid conditions, charlson comorbidity index, vascular access, and class number of antihypertensive agents. Abbreviations: DM, diabetes mellitus; CVD, cardiovascular disease; BMI, body mass index. (TIFF) [file pone.0235900.s005.tiff]
